# Surface-antigen expression profiling of B cell chronic lymphocytic leukemia: from the signature of specific disease subsets to the identification of markers with prognostic relevance

**DOI:** 10.1186/1479-5876-4-11

**Published:** 2006-03-01

**Authors:** Antonella Zucchetto, Paolo Sonego, Massimo Degan, Riccardo Bomben, Michele Dal Bo, Pietro Bulian, Dania Benedetti, Maurizio Rupolo, Giovanni Del Poeta, Renato Campanini, Valter Gattei

**Affiliations:** 1 Clinical and Experimental Hematology Research Unit, Centro di Riferimento Oncologico, I.R.C.C.S., Aviano (PN), Italy; 2 Medical Oncology, Centro di Riferimento Oncologico, I.R.C.C.S., Aviano (PN), Italy; 3 Chair of Hematology, University of Tor Vergata, S. Eugenio Hospital, Rome, Italy; 4 Department of Physics, University of Bologna, INFN-Sezione di Bologna, Bologna; Italy

## Abstract

Studies of gene expression profiling have been successfully used for the identification of molecules to be employed as potential prognosticators. In analogy with gene expression profiling, we have recently proposed a novel method to identify the immunophenotypic signature of B-cell chronic lymphocytic leukemia subsets with different prognosis, named surface-antigen expression profiling. According to this approach, surface marker expression data can be analysed by data mining tools identical to those employed in gene expression profiling studies, including unsupervised and supervised algorithms, with the aim of identifying the immunophenotypic signature of B-cell chronic lymphocytic leukemia subsets with different prognosis. Here we provide an overview of the overall strategy employed for the development of such an "outcome class-predictor" based on surface-antigen expression signatures. In addition, we will also discuss how to transfer the obtained information into the routine clinical practice by providing a flow-chart indicating how to select the most relevant antigens and build-up a prognostic scoring system by weighing each antigen according to its predictive power. Although referred to B-cell chronic lymphocytic leukemia, the methodology discussed here can be also useful in the study of diseases other than B-cell chronic lymphocytic leukemia, when the purpose is to identify novel prognostic determinants.

## Background

B-cell chronic lymphocytic leukemia (B-CLL) is a heterogeneous disease with highly variable clinical courses. Two major clinical staging systems, mainly based on tumor load, were developed to estimate prognosis in B-CLL [1-3]. Both these systems, however, are unable to prospectively discriminate between the rapidly evolving patients from those destined to remain with a stable disease for decades. Therefore, continuous efforts have been produced to identify additional prognostic factors, which may help to better define patient cohorts with different clinical outcome.

The mutational status of IgV_H _genes has recently been identified as a robust indicator of disease outcome: patients with a disease characterized by neoplastic cells bearing a mutated IgV_H _gene configuration had significantly longer survival than those cases affected by B-CLL expressing unmutated IgV_H _genes. Since IgV_H _mutation testing is an expensive and technically difficult assay not widely applicable for clinical use, subsequent studies were focused on the identification of alternative markers with prognostic value similar to that of IgV_H _mutations, and whose expression could easily be investigated, e.g. by flow cytometry. Several reports identified the over-expression of CD38 as a marker of poor prognosis for B-CLL patients [4]. However, the cut-off values of CD38 expression capable to segregate B-CLL patients into groups with different survivals varied in some studies [4-6]., and the expression of CD38 over a given threshold failed to maintain a statistically significant correlation with survivals by multivariate analysis [7]. Moreover, the capability of CD38 to act as a surrogate of IgV_H _mutational status, initially emphasized [8], was not confirmed by subsequent reports [4-6,9].

Studies of gene expression profiling (GEP) have been successfully used for the identification of additional molecules to be employed as potential prognosticators [10-13]. Among them, the gene encoding for the T cell specific zeta-associated protein 70 (ZAP-70) has been demonstrated to have both a prognostic relevance and a predictive power as surrogate for IgV_H _mutations [10,14-16]. The detection of the ZAP-70 gene product by flow cytometry, however, is not easy to be performed, since it requires cell membrane permeabilization and the simultaneous use of T cell markers to discriminate the expression of ZAP-70 protein between malignant B-CLL cells and residual T lymphocytes [14].

In analogy with GEP, we have recently proposed a novel method to identify the immunophenotypic signature of B-CLL subsets with different prognosis, named surface-antigen expression profiling (SEP)[17,18]. In our original proposal, the expression of a wide panel of surface markers was analysed in a cohort of 123 B-CLLs with known survivals, by means of data mining tools identical to those employed in GEP studies [17,19-21]. By sequentially applying unsupervised (hierarchical and non-hierarchical) clustering algorithms, and the nearest shrunken centroid method as class predictor, we were able to identify the signature of three subsets, one corresponding to good prognosis B-CLLs, and two identifying subgroups with shorter survivals [17].

We will provide here an overview of the strategy employed for the development of this sort of "outcome class-predictor" for B-CLL based on surface-antigen expression. In particular, we will discuss how to compute flow cytometry data, the rationale for the choice of sequential unsupervised/supervised analyses eventually yielding to the signature of the identified disease subsets. Finally, we will also discuss how to transfer the information gained through the proposed class-predictor into the routine clinical procedures to refine the identification of B-CLL patients with different prognosis. In particular, we will summarize a flow-chart indicating how to select the immunophenotypic markers with the most relevant prognostic impact and how to build-up a prognostic scoring system by giving different weights to each antigen according to its predictive power. Part of the data extensively discussed in this section of the present review has been recently reported by our group [22].

All the comments and analyses reported below, although referred to B-CLLs, can be useful to transfer a similar approach into the study of diseases other than B-CLL when the aim is of identifying novel prognostic determinants.

## Generation of flow cytometric data

Expression of surface antigens is usually investigated by multicolor flow cytometry [23]. In our reports on B-CLL [17,18]., we investigated the expression of a wide panel of surface markers by two- or three-color flow cytometry, combining phycoerithrin (PE)-, fluorescein isothyocyanate (FITC)- and allophycocyanin (APC)-conjugated monoclonal antibodies (mAbs) [3]. Results of antigen expression were always reported as per cent of CD19^+ ^B-CLL cells displaying a specific fluorescence intensity greater than the 98–99% of the same cell population stained with isotype- and fluorochrome-matched control immunoglobulins [8,17,24,25]. It is in our opinion that this choice, although not the optimum in absolute terms, represents nowadays a relatively good tool with some undoubted advantages as compared to mean fluorescence intensity (MFI) or other equivalent absolute measurings [26]:

- advantages for the subsequent analyses: in our hands the use of percent of positive cells allowed a better application of the methods of cluster analyses reported below; this happens basically for two reasons: i) it facilitates clustering by decreasing the complexity of the final database (a marker expressed above the threshold corresponding to 100% of positive cells will be always considered expressed at the value of 100); ii) it reduces the operational range of the color scale employed in heat maps (0–100 in all cases).

- advantages for the recruitment of cases: an absolute measuring of flow cytometry data, e.g. by converting MFI values into molecules equivalent of soluble fluorochrome (MESF) [26], can be made only through the use of specific calibration beads run in parallel during each single experiment; this greatly limits, if not forbids, any retrospective analysis of flow cytometry data (for example data generated for diagnostic purposes in clinical-oriented laboratories).

- advantages due to the characteristics of the chosen panel: the use of percent of positive cells as a measure of antigen expression allows to better compare all the employed antigens to each other especially when the panel of interest: i) includes mAbs conjugated with fluorochromes with different relative brightness (FITC, PE, APC); ii) includes a certain number of unlabeled mAbs, which, by requiring additional binding with secondary fluorochrome-conjugated antibodies, usually yield higher MFI values than directly fluorochrome-conjugated mAbs; iii) includes mAbs recognizing phenotypic markers known to have greatly different cellular density as compared to others (e.g. CD24, CD52 and CD59) [17,18,27].

Last but not least, this choice of analysis might be prospectively more useful in the field of B-CLL for two additional reasons: i) compare the expression of some markers of supposed prognostic significance with that of other well-established prognosticators, e.g. CD38 or ZAP-70, whose expression level is usually reported as percent of positive cells [8,10,14-16,24,28]; ii) as underscored below, since the final aim is to build-up a scoring system of clinical relevance, the evaluation of the expression level for each selected marker as per cent of positive cells allows an easier application of the scoring system in the context of diagnostic routine laboratories.

## Analyses of flow cytometric data with unsupervised/supervised data mining tools

If the working hypothesis is to analyse surface antigen expression data in order to identify disease subsets characterized by a given expression pattern, we need computational tools capable to simultaneously compare expression levels of the various antigens among different cell samples.

In our B-CLL studies [17,18]., flow cytometric data, generated as reported above, have been analysed by taking advantage of the unsupervised/supervised algorithms publicly available with the PAM (prediction analysis for microarray) statistical package [19,20,29] and through the open source versions of the Cluster and TreeView programs [30,31].

The chosen statistical and computational tools of pattern recognition/classification are those normally employed in GEP studies [19,20]. So far, a similar approach to analyse flow cytometry data has been only sporadically utilized, e.g. to investigate antigen expression profiles of myeloid cell subsets in myelodysplastic syndromes [32] or of blast cells in childhood lymphoblastic leukemias [26], as well as by us in preliminary studies of immunophenotypic clustering of B-CLL cells [33]. On the other hand, cluster analysis and dendrograms have been historically employed in the context of "International Workshops of Leukocyte Typing" to define the reactivity of specific monoclonal antibodies and identify whether they recognize the same or closely related molecules, thus defining novel "clusters of differentiation" (CDs) [34]. In all these reports, however, antigen expression profiles were exclusively analysed by means of unsupervised methods, such as hierarchical clustering [32-34] or principal component analysis [26,32]., by definition not taking into account any additional external factors, such as survivals or other clinical signs. If the aim is of defining the immunophenotypic signatures of disease subsets with specific clinical features, e.g. a different prognosis, it is mandatory to perform a so-called "supervised" analysis, that, conversely, does take into account specific, pre-defined, external factors [21,35].

**Figure 1:**
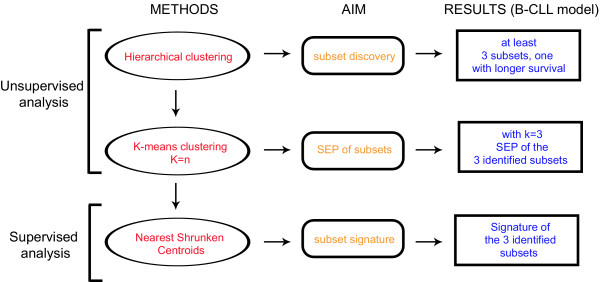
**From the hierarchical clustering to the immunophenotypic signature of subsets with different prognosis**. See text for details.

In our original proposal on B-CLL [17], we have employed, along with unsupervised clustering algorithms, the supervised method of the "nearest shrunken centroids" as class predictor to discover the antigen expression patterns associated with a given prognosis (see below). In preliminary analyses, by taking advantage of survival data, we tried to directly apply a supervised learning procedure to distinguish stable from progressive/fatal diseases, basically as described for diffuse large B cell lymphomas using cDNA microarray [21]. This approach, however, failed to define a reliable surface antigen-expression signature capable to distinguish B-CLLs with different clinical courses, e.g. stable vs. progressive diseases [17]. Therefore, we decided for a strategy, summarized in Fig. 1, in which, the application of unsupervised (hierarchical and non-hierarchical) clustering methods, was utilized to identify the minimum number of phenotypic clusters associated with differences in a specific clinical feature (in our case survival); these subsets were then characterized for the antigens differentially expressed by taking advantage of supervised algorithms, as detailed below.

## Unsupervised analyses of immunophenotypic data: from the identification of the smallest number of subsets with different prognosis to their surface-antigen expression profiling (SEP)

Operationally, in our original report [17], we analysed a complete database containing the expression values of 36 surface antigens in 123 B-CLL samples (4428 theoretical values) by an unsupervised hierarchical clustering choosing a complete-linkage method and the euclidean distances as measures of similar/dissimilar behaviour [17-19]. Survivals of the resulting B-CLL clusters were tested using Kaplan-Meier analysis and log-rank test [36]. As shown in [17], hierarchical clustering allowed the discovery of at least three different B-CLL subsets, one of them with strikingly better prognosis as compared to the others (Fig. 1). Subsequently, we re-analysed expression data by applying a k-means clustering, i.e. a non-hierarchical unsupervised method which allows partitioning of data into predetermined (k) groups [37-39]. In agreement with the results of hierarchical clustering, we applied a k-means clustering algorithm by requiring a separation of all B-CLL cases into three subgroups (k = 3) (Fig. 1) [17].

The sequential use of hierarchical clustering and k-means to eventually define the SEP of a given number of disease subsets (in our example three B-CLL subsets) has its rationale in the specific mathematical algorithms underlying these two unsupervised methods. In particular, hierarchical clustering is usually utilized in preliminary screening, when no further information is available; in our example, hierarchical clustering of B-CLLs, if associated with Kaplan-Meier analysis [36], was able to give us the information that: (i) there is correlation between survival and immunophenotypic profile; (ii) at least three clusters can be associated with a different prognosis (Fig. 1) [17]. Once obtained this information, k-means is applied as an additional unsupervised algorithm capable to split a given data-set into a certain number of clusters fixed a priori (the assumed k clusters). Obviously, this method can be applied only when there is a supported hypothesis suggesting the number of clusters in which a given data-set has to be split. As opposed to hierarchical clustering, that defines the "distances" among the various clusters, the algorithm of k-means, by emphasizing analogies instead of differences, is optimal for the purpose to subdivide a data set into few pre-established subsets.

The three B-CLL clusters identified by the sequential application of hierarchical and k-means analyses (named group I, II and III in [17]) were prognostically characterized by different survivals: longer for group I patients (51 cases all alive but one case, with a maximal follow-up of about 250 months), shorter for groups II and III patients (overall accounting for 72 cases, median survivals of about 100 months for both groups); although displaying similar survivals, B-CLL cells from patients belonging to these two latter groups had different immunophenotypes [17]. The complex immophenotypic profiles characterizing each group have been reported in extenso elsewhere [17]. Here we solely mention that specific sets of antigens appeared overexpressed or downregulated in the context of the various subgroups; the subsequent supervised analyses (see below) allowed the identification of the few markers really representing the "signature" of each prognostic group.

## Supervised analysis of immunophenotypic data by shrunken centroids: from the surface-antigen expression profiling (SEP) of subsets with different prognosis to their immunophenotypic signature

In our original studies, the combination of unsupervised analyses (hierarchical and k-means) and Kaplan-Meier survival curves, allowed the identification of three phenotypic clusters in B-CLLs, one corresponding to a good prognosis B-CLL subset, the remaining two clusters characterized by shorter median survivals, although with different immunophenotypic profiles [17].

The next logical step is the identification of the phenotypic signature, i.e. the minimal number of surface markers succinctly characterizing each B-CLL prognostic group. For this purpose, we chose to apply the "nearest shrunken centroid" algorithm, as proposed by Tibshirani et al [17,20]., utilized by taking advantage of the publicly available PAM software package [19,20]. This method basically derives from the "nearest centroid" classification. Briefly, the "nearest centroid" method computes a standardized centroid for the expression values of a given antigen in a given subgroup. A "centroid" has to be intended as a measure of the average expression of a given antigen in a given set/subset of samples. The "nearest centroid" classification method takes the antigen expression level of a new sample and compares it to each of these centroids. The subgroup whose centroid is closest to, is the predicted subgroup for that new sample.

The "nearest shrunken centroid" classification makes one important modification to standard "nearest centroid" classification. For a given antigen, it shrinks each centroid corresponding to each subgroup toward the overall centroid (i.e. the centroid computed by considering all the subgroups) by a fixed amount, called "threshold". This shrinkage consists of moving the centroid towards zero by a value corresponding to the threshold, setting it equal to zero if it hits zero. After shrinking the centroids, the new sample is classified by the usual nearest centroid rule, but using the shrunken centroids. This shrinkage has the operational advantages (i) to make the classifier more accurate by reducing the effect of noisy antigens, and (ii) to do an automatic selection of the antigens. In particular, if a given antigen is shrunk to zero for all the subgroups, then it is eliminated from the prediction rule. Alternatively, it may be set to zero for all subgroups except one; in this latter case, we learn that above- or below-average expression for that antigen characterizes that subgroup.

In the "nearest shrunken centroid" method, the user decides on the value for threshold to be employed. Typically, different choices are examined, and to guide the user to this choice, PAM performs k-fold (where k is usually set at the value of 10) cross-validation (CV) for a range of threshold values. Basically, a model is fitted on 90% of the sample and the class of the remaining 10% is predicted; this procedure is repeated 10 times, with each sample being part of the 10% utilized as tester at least once. The overall error is obtained by summing the errors of all the 10 parts added together. This procedure is done for a series of threshold values, and the number of CV misclassification errors for each threshold level allows the user to choose the value giving the minimum cross-validated misclassification error rate.

In the case of our original B-CLL studies, we randomly divided immunophenotypic data (123 cases) into training data (90 cases) and test data (33 cases) [17]; data from the 90 training samples were employed to train a classifier by means of 10-fold CV, while test samples were utilized as validation of the found procedures [17,20]. In particular, we selected three increasing values of threshold (0.66, 1.32 and 2.0), all corresponding to acceptable misclassification errors on cross-validated data. By increasing the threshold level, the minimum number of antigens correctly classifying a given B-CLL case shrank in parallel. At the highest acceptable threshold level (2.0), close to the point at which CV error started to rise [17], as low as 12 phenotypic markers were selected [20]. As reported [17], the estimated probabilities to classify B-CLL training and test samples within a given group (i.e. I, II or III) by using the threshold value of 2.0 were fairly good for training samples, less for test samples (e.g. 10-fold CV errors = 6/90; test error = 6/33). The accuracy of sample classification increased by lowering the threshold, although, in these cases, the number of essential phenotypic markers increased up to 18 (threshold = 1.32) and 23 (threshold = 0.66) antigens, always including the 12 markers selected by setting the threshold at the highest acceptable value (2.0) [17].

A list of the 12 selected markers overall representing the immunophenotypic signature of the three B-CLL subsets with different prognosis are reported in Fig. 3. Briefly, the good prognosis B-CLL group I was characterized by the specific above-average expression of CD62L, CD54, CD49c and CD25. Among B-CLL cases with shorter survival (groups II and III), two clearly recognizable immunophenotypic patterns were identified: the first one, with above-average expression of CD38, CD49d, CD29 and CD49e, was specific for group II B-CLLs, whereas group III was characterized by the expression below-average of all the above reported markers in the presence of a relative overexpression of some common antigens, such as CD23, CD20, SmIg and CD79b (Fig. 3) [17].

Even though information regarding correlations with IgV_H _mutations and ZAP-70 expression as well as the biological implication underlying differential expression of critical molecules in the newly identified B-CLL subsets have been extensively discussed elsewhere [17], we briefly report herein the most relevant concepts about this matter.

## IgV_H _mutational status and ZAP-70 expression in the three immunophenotypic groups

IgV_H _mutations and ZAP-70 expression have been reported to be among the most important prognosticators for B-CLL [5,8,14-16,28,40-42]; in this regard IgV_H _mutations below the established cut-off of 2% and expression of ZAP-70 in more than 20% of neoplastic cells had a negative impact on survivals also in our series [17]. Consistently, patients whose neoplastic component displayed a mutated IgV_H _gene configuration or expressed ZAP-70 in less than 20% of cells frequently belonged to the immunophenotypic group characterized by the best prognosis (group I); conversely, patients characterized by B-CLL cells lacking IgV_H _mutations or with high ZAP-70 expression levels belonged more frequently to the immunophenotypic groups associated with worse prognosis (groups II and III) [17].

## Biological meaning of the different immunophenotypic profiles

According to the presented results, overexpression of CD62L, CD54, CD49c and CD25 in the absence of CD38 represented the immunophenotypic signature of good prognosis B-CLL. Noteworthy CD62L, together with CD54 and CD25 are all surface structures somewhat involved in the cross-talk between B lymphocytes with neighbouring endothelial and/or T cells within the lymph node microenvironment [43-47]. Interestingly, B-CLL cases expressing the CD62L^+^CD54^+^CD25^+ ^phenotype more frequently displayed high number of IgV_H _mutations [5,8,28,40-42], as well as an IgV_H _mutational status consistent with antigen-driven selection [25,48,49]. These data are in keeping with the immunophenotypic profile of these cells, since IgV_H _mutations usually occur as the result of T cell-dependent interactions during GC maturation of B cells [50].

One of the two immunophenotypic profiles characterized by worse prognosis (group II) was distinguished by the above-average expression of CD38, CD49d, CD29 and CD49e. This finding is in keeping with recent reports in which high levels of CD49d mRNA and protein were found to be part of the signature distinguishing CD38^+ ^from CD38^- ^B-CLL cells [51,52]. The above-average expression of CD49d in B-CLL cases with a poorer prognosis is also consistent with the demonstration of higher expression levels of this molecule in B-CLL cells from advanced stage patients [53], as well as with the notion that engagement of α4β1, as expressed by B-CLL cells, triggers a signalling cascade eventually preventing apoptosis [54-56]. Along with CD49d and CD38, other negative prognosticators, including ZAP-70 and a low IgV_H _mutation load [8,14-16,28,40], were more frequently found in group II B-CLLs.

A third immunophenotypic profile (group III), associated with poor prognosis and succinctly characterized by the below-average expression of all the markers reported above as over-expressed in group I (CD62L, CD54, CD25 and CD49c) or group II (CD38, CD49d, CD29 and CD49e) B-CLLs, was revealed by supervised analysis [17]. The identification of this B-CLL subgroup is somewhat surprising, since these cases represent a poor prognosis subset essentially lacking CD38 expression and without an excess of cases with low IgV_H _percent mutations and/or lack of antigen-driven selection [28,48,49]; this group might, therefore, represent a separate entity, allegedly characterized by a distinct pathway of oncogenesis [6,57])), that deserves to be further investigated. If confirmed in larger cohorts of patients, the identification of this group as a genuine biological subset of B-CLLs may contribute to explain some discrepancies found in the recent literature, such as the definition of the optimal cut-offs for CD38 expression and percent IgV_H _mutations capable to split B-CLL patients into subgroups with different survivals [5,42].

## From the signature of subsets with different prognosis to the identification of novel determinants with prognostic value

As summarized in Fig. 3, the coordinated above- and/or below-average expression of 12 surface antigens characterizes the immunophenotypic signature of three B-CLL groups with different prognosis.

In the present paragraph, we discussed the overall strategy allowing the selection, among the markers identifying each subset, of those with the most relevant prognostic impact to be eventually used for prognostic purposes in clinically-oriented laboratories. Such a strategy included at least two initial steps aimed (i) at reducing the number of prognosticators by keeping only the most relevant of them, and (ii) at finding the optimal cut-off points to be employed to discriminate cases expressing or not a given marker. In the case of B-CLLs, we operated as follows:

### i) Identification of the antigens with the highest statistically significant independent predictive power

This first point was addressed by applying the Cox proportional hazards regression model on expression data for the 12 antigens with overall survival as dependent variable [58]. The notion that all the investigated markers derived from a previous clustering, made their expression levels, at least to a certain extent, each other interrelated; therefore, a multivariate analysis, that, by definition, can compare only variables with an independent behaviour [59,60]., was not suitable for correlating antigen expression values and survivals in our series. We therefore chose to perform, instead of a multivariate analysis, an univariate analysis.

The Cox proportional hazards regression model computes a coefficient (z score) for each predictor marker that indicates the direction and degree of flexing that the predictor has on the survival curve. Zero means that a given marker has no effect on the curve, i.e. it is not a predictor at all; a positive z score indicates that larger values of the marker are associated with greater mortality, while a negative z score indicates that larger values of the variable are associated with lesser mortality.

A list of the actual z scores, as found by us in a series of 137 B-CLLs [22] are reported in Fig. 3. According to this analysis, we were able to select six antigens displaying a z score with an absolute value of 2.0 or greater (p < 0.05). These markers were CD62L, CD54, CD49c, associated with a negative z score, therefore identified as positive prognosticators, and CD49d, CD38, CD79b, associated with a positive z score, hence recognized as negative prognosticators (Fig. 3).

### ii) Estimation of antigen expression levels yielding the best separation of two subsets with different survival probabilities

Once identified the phenotypic markers with greater prognostic impact, we operated to find, for each of them, the optimal cut-off value yielding the best separation between two subgroups with different survival probabilities.

In general it can be assumed that any given prognosticator (in our case, expression values for a given antigen) allows for a classification of patients into groups with different risk with respect to a response variable (e.g. death, progression of disease etc.). The functional relationship between the putative prognosticator and the response variable is unknown. Any given cut-off point for the expression level of an antigen determines two groups of patients, i.e. a group with all the patients in which the variable is less or equal to a given cut-off point, and a group of patients in which the variable is greater than the same cut-off point. The determination of the optimal cut-off point for each antigen yielding the best separation between two subgroups with different survival probabilities was achieved by applying the maximally selected log-rank statistics [61], available as open source program at the reported website [62].

**Figure 2:**
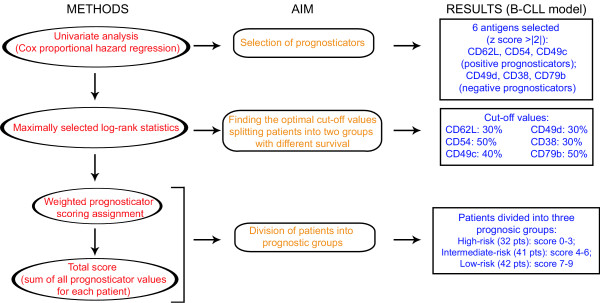
**From the signature of subsets with different prognosis to the definition of a comprehensive prognostic scoring system and the division of patients into prognostic groups**. See text for details.

**Figure 3:**
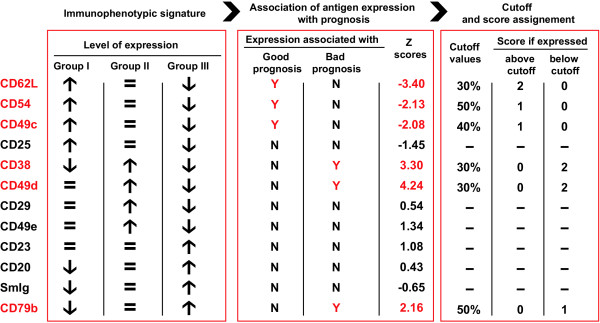
**Surface antigens identified by the nearest shrunken centroid algorithm and their role as putative prognosticators for B-CLLs**. For each antigen identified as part of the immunophenotypic signature, are here reported: i) its expression levels in the three immunophenotypic groups as identified by nearest shrunken centroids (↑ or ↓ indicate above- or below-average expression, respectively; = indicates average expression level) [17]; ii) its association (Y) or lack of association (N) with good or bad prognosis and the relative z scores as resulted by Cox proportional hazard regression analysis [22]; iii) the cutoff values, as resulted by Maximally selected log-rank statistics analysis, and the score values assigned when its expression was found to be above or below the established cutoff [22]. Data and values of the antigens selected for the final prognostic score are reported in red.

As reported in Figs. 2 and 3, the application of this statistical algorithm allowed to find different optimal cut-off values for each of the six antigens previously selected by univariate analysis [22]. This approach, although not yet widely applied, has been found useful by us and others in the identification of the most suitable cut-off points for CD38 and IgV_H _mutations in studies aimed at testing the strength of these markers as prognosticators [5,63,64].

## Comprehensive prognostic score and division of patients into prognostic groups

Taken together, univariate Cox proportional hazard regression and maximally selected log-rank statistics provide useful information on the prognostic value of each single antigen, when considered alone. As a next step, we tried to improve the prognostic assessment of B-CLL by combining the expression values of the six antigens into a comprehensive prognostic score. To do this, we operated as follows (Figs. 2 and 3):

### i) Assignment of a different statistical weight to each selected antigen

Scores of "0", "1" and "2" were assigned to each prognosticator according to the z score found for each antigen and the expression above or below the established cut-off value. In particular, for positive prognosticators (CD62L, CD49c and CD54) a score "0" was assigned when the expression was below the cut-off values, while for negative prognosticators (CD49d, CD38 and CD79b) a score "0" was assigned when the expression was above the established cut-offs. Scores "1" were assigned to the positive prognosticators CD49c and CD54 when the expression was above the established cut-offs, and to the negative prognosticator CD79b when the expression was below the established cut-off. These markers were characterized by z scores comprised between the absolute values of 2 and 3 (Fig. 3). Scores "2" were assigned to the positive prognosticator CD62L when the expression was above the established cut-off, and to negative prognosticators CD49d and CD38 when their expression was below the established cut-offs. These markers were characterized by z scores exceeding the absolute value of 3 (Fig. 3).

A similar approach, in which the expression and/or the presence of specific markers are evaluated with different statistical weights according to given established parameters, has been already employed to define similar diagnostic/prognostic scoring systems in onco-hematology [47,65,66]. Fig. 3 summarizes the score values associated with each single prognosticator.

### ii) Computation of a total score and division of patients into prognostic groups

The final step is the sum of the values found for each prognosticator by considering the assigned scores (0,1 or 2), as defined above. In a series of 115 B-CLL patients we obtained total score values ranging from "0" (complete absence of phenotypic conditions associated with good prognosis) up to "9" (all the phenotypic conditions associated with good prognosis fulfilled). Overall, the 115 B-CLL patients showed a median survival time of 157 months with 95% confidence intervals ranging from 120 months to "not reached" [22]. The same B-CLL patients, when ranked according to their total score, could be divided into three, roughly quantitatively homogeneous, groups: score 0–3 (32 cases); score 4–6 (41 cases), and score 7–9 (42 cases) [22]. According to Kaplan-Meier survival probabilities, there was significant difference among the three groups (p = 4.78 × 10^-11^by the log-rank test). We therefore labeled the identified three prognostic groups as high-, intermediate- and low- risk groups, respectively.

**Figure 4:**
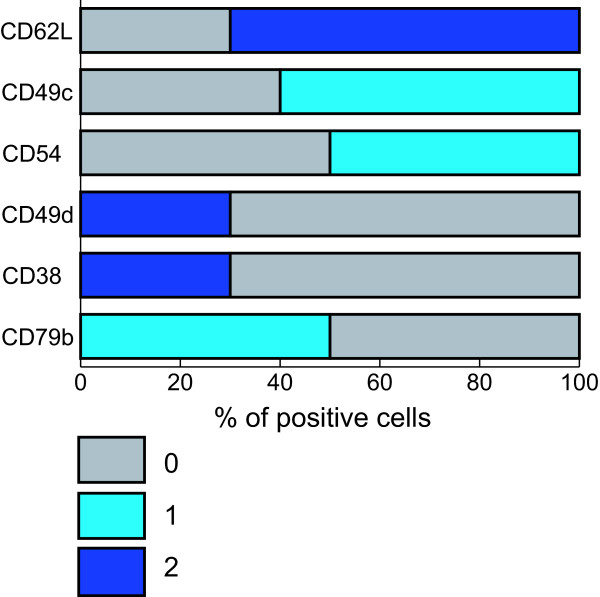
**Translation of the prognostic values for the identified six antigens into a scoring system**. Each of the six antigens previously identified as the most powerful prognosticators [17,22] was associated with a score of 0, 1 or 2 according to its predictive power (z score) and the expression above or below the established cut-off values. Each of the reported bar, corresponding to the theoretical expression values (reported as % of positive cells) for each antigen, is depicted in grey (score "0" zone), azure (score "1" zone) or blue (score "2" zone). See text for detailed discussion of score assignment and cut-off values.

Fig. 4 represents an operational scheme for rapidly applying the prognostic scoring system proposed by us in a clinical setting. Briefly, the six selected prognosticators are indicated by bars corresponding to the theoretical expression values reported as per cent of positive cells; each bar is depicted in different colors according to the score "0", "1"or "2" zones (Fig. 4).

## Conclusion

By summarizing some recent studies by our group [17,18,22], we discussed here a general strategy for the application of data mining tools usually employed in GEP to analyse flow cytometry data in the field of B-CLL. By sequentially applying unsupervised algorithms (hierarchical clustering and k-means) and the nearest shrunken centroid method as class-predictor, we have been able to identify a number of surface antigens characterizing specific immunophenotypic subsets, each of them associated with different survivals. We also discussed a stepwise approach for selecting few immunophenotypic prognosticators and integrating them in a specific scoring system capable to provide a more refined prognostic assessment than that relying on the evaluation of the presence/absence of any single prognosticator. As an example, if we had solely considered the distribution of the negative prognosticators CD38 or ZAP-70 [8,11,14-16,24,28,67-71] in the three B-CLL risk groups identified by applying this novel scoring system [17,22]., almost one/third of patients would have been misclassified regarding their prognosis. Similar discrepancies have been recently underscored by us and others in reports demonstrating that the predictive power associated with the combined detection of CD38 and ZAP-70 [72], or of CD38 and CD49d [52] was more precise than that associated with the single factors.

The approach proposed and discussed in the present review, although referred to B-CLL, may have a more general interest, being easily applicable to diseases other than B-CLL with the aim of identifying novel prognostic determinants.

## Competing interests

The author(s) declare that they have no competing interests.

## Authors' contributions

AZ carried out immunophenotypic studies of the original reports and contributed to write the draft of the present manuscript; PS and RC made substantial contribution in the analyses and interpretation of data reported in the original studies and contributed in writing the statistical paragraphs of the present manuscript; MD, RB, MDB and DB performed all the molecular analyses, contributed to the generation of immunophenotypic data of the original reports, and partially contributed to write the present draft; PB, MR and GDP made B-CLL diagnoses and collected clinical data of patients of the original reports; VG coordinated all the original studies, participated in the analyses and interpretation of data and wrote the present manuscript.
